# Relationship between endothelial activation and stress index and all-cause mortality in rheumatoid arthritis patients: a moderating effect of gamma-glutamyl transferase

**DOI:** 10.3389/fnut.2025.1554429

**Published:** 2025-04-23

**Authors:** Wenjing Chang, Zhiming Zhao, Linlin Ma, Le Lu, Chunli Liu, Mengdi Hu, Wei Shang

**Affiliations:** ^1^Department of Traditional Chinese Medicine, Affiliated Jinling Hospital, Medical School of Nanjing University, Nanjing, China; ^2^Department of Clinic, Affiliated Jinling Hospital, Medical School of Nanjing University, Nanjing, China

**Keywords:** endothelial activation and stress index, gamma-glutamyl transferase, all-cause mortality, rheumatoid arthritis, moderating effect

## Abstract

**Aim:**

This study aimed to explore the relationship between endothelial activation and stress index (EASIX) and all-cause mortality in patients with rheumatoid arthritis (RA), and to further examine whether gamma-glutamyl transferase (GGT) influences this association.

**Methods:**

We included 2,543 participants with RA from the National Health and Nutrition Examination Survey (NHANES) in this retrospective cohort study. The study outcome was considered to be all-cause mortality. EASIX and GGT levels were measured at baseline (study enrollment) using laboratory data from NHANES. EASIX was divided into two groups based on its median: ≥0.476 and <0.476, while GGT was divided into two groups based on its median: ≥23 U/L and <23 U/L. EASIX was calculated using the formula, lactate dehydrogenase (LDH, U/L) × creatinine (mg/dL)/platelet count (10^9^/L), based on the baseline laboratory measurements. Weighted multivariate Cox regression models were used to assess the associations between EASIX and GGT with the risk of all-cause mortality. Importantly, a moderated analysis of GGT (moderator) was conducted to examine the relationship between EASIX and all-cause mortality among patients with RA. Additionally, subgroup analysis was performed based on age, duration of arthritis, diabetes, and hypertension.

**Results:**

A total of 867 individuals developed all-cause mortality over a mean follow-up period of 122.86 ± 3.29 months. After fully adjusting for potential confounding factors, higher EASIX (≥0.476) was positively associated with all-cause mortality (hazard ratio [HR] = 1.42; 95% confidence interval [CI]: 1.18–1.73). However, the association between GGT and all-cause mortality was not significant (*p* > 0.05). Moderated analysis revealed that higher GGT levels strengthened the correlation between EASIX and all-cause mortality among patients with RA (*p* = 0.013). The association between EASIX and the risk of all-cause mortality varied depending on GGT levels. The subgroup analysis revealed that GGT moderated the relationship between EASIX and all-cause mortality among RA patients aged 60 years or older (*p* = 0.007), with a history of arthritis lasting more than 5 years (*p* = 0.040), or diagnosed with diabetes (*p* = 0.009) or hypertension (*p* = 0.016). Competing risks analysis accounting for cardiovascular mortality yielded consistent results (subdistribution hazard ratio [sHR] = 1.39; 95% CI: 1.15–1.69), further supporting the primary findings.

**Conclusion:**

High EASIX was positively associated with all-cause mortality in patients with RA, and this association was significantly enhanced by higher GGT levels.

## Introduction

Rheumatoid arthritis (RA) is a chronic, systemic, inflammatory autoimmune disease that may lead to permanent disability and a higher mortality rate (1). In 2020, the global prevalence of RA was estimated at 17.6 million cases, with projections indicating an increase to 31.7 million individuals affected by 2050 (2). Patients diagnosed with RA have a decreased life expectancy, and the study found that they exhibited a 54% higher risk of all-cause mortality compared to the general population (3). Understanding the factors that influence RA mortality is crucial for optimizing management approaches and intervention strategies.

Inflammation may be considered an important factor leading to premature death in RA patients (4). Research indicates that approximately 25% of all-cause mortality in RA patients can be linked to the effects of inflammatory mediators (5). Endothelial cells serve as both active participants and regulators in the inflammatory process (6). In patients with RA, activated endothelial cells are prevalent and have the potential to contribute to the development of atherosclerosis (7). Assessing endothelial function may prove to be a valuable tool for identifying and monitoring individuals with RA (8). Recently, a new index for evaluating endothelial injury and dysfunction, the endothelial activation and stress index (EASIX), has been proposed and utilized to predict the prognosis of critically ill patients (9) and those with cancer (10). However, the impact of EASIX on the prognosis of patients with RA remains uncertain. In addition, the imbalance between reactive oxygen species and antioxidant systems, known as oxidative stress, is the primary factor influencing the development of endothelial dysfunction and contributing to metabolic and cardiovascular diseases (CVD) (11). The relationship between oxidative stress and endothelial dysfunction has also been observed in patients with RA (12). Gamma-glutamyl transferase (GGT) is an indicator of liver function and has recently been increasingly utilized as a reflection of the body’s oxidative balance (13, 14). Elevated GGT levels were associated with endothelial dysfunction in patients diagnosed with non-alcoholic steatohepatitis (15) and an increased risk of mortality in the general population (16). Vergneault et al. found that GGT may serve as a marker of systemic inflammation and CVD risk in patients with RA (17). However, there has been limited research investigating the potential impact of GGT levels on mortality risk associated with endothelial dysfunction in individuals with RA.

Herein, the present study utilized data from the National Health and Nutrition Examination Survey (NHANES) database to explore the relationship between EASIX and the risk of all-cause mortality in patients with RA, as well as to further examine whether GGT levels influence this association.

## Methods

### Study population

In this retrospective cohort study, all data were obtained from the NHANES database. NHANES is a complex, multistage, and probabilistic sampling design survey that collects information from participants through interviews (demographic, socioeconomic, dietary, and health-related questions) and physical examinations (medical, dental, physiological measurements, and laboratory tests) (18). The protocol of NHANES was approved by the National Centre for Health Statistics (NCHS) ethics review board.

A total of 2,952 individuals aged 20 years or older with RA were identified from the NHANES data collected between 1999 and 2018. Baseline refers to the time of NHANES enrollment, rather than the RA diagnosis. All measurements were taken during the initial participant evaluation. The diagnosis of RA was obtained through a self-report questionnaire (19), where participants were asked the question “Has a doctor or other health professional ever told you that you had arthritis?” The response options included “Yes” or “No.” RA was assessed using the following question: “Which type of arthritis was it?” The response options were “RA,” Osteoarthritis,” “Psoriatic arthritis,” “Other,” “Refused,” and “Do not know.” We excluded some individuals with incomplete measurements of GGT (n = 336) or missing data for calculating EASIX (*n* = 21). Additionally, individuals without survival status were further excluded (*n* = 52). The survey year was included as a covariate to account for temporal trends in RA management. Stratified analyses by treatment era (pre-/post-2010) revealed no significant interactions. Finally, 2,543 subjects were enrolled in this study.

### Data collection

#### Outcome

The primary outcome considered in this study was all-cause mortality. All-cause mortality was determined using the records from the National Death Index (NDI) up to 31 December 2019, which were linked to the NHANES datasets.

#### Assessment of EASIX and GGT

All laboratory parameters (LDH, creatinine, platelet count, and GGT) were measured at baseline enrollment in NHANES. These values represent a single timepoint assessment at study entry and were not longitudinally tracked during follow-up. The EASIX score was calculated using the formula: lactate dehydrogenase (LDH, U/L) × creatinine (mg/dL)/platelet count (10^9^/L) (1). In this study, EASIX was divided into two groups based on the median: ≥0.476 and <0.476.

GGT data were obtained from the NHANES laboratory tests. The NHANES participants provided a blood specimen for laboratory analyses. GGT concentration was measured using a Hitachi Model 704 multichannel analyzer (Boehringer Mannheim Diagnostics, Indianapolis, IN). GGT was divided into two groups based on median: ≥23 U/L and <23 U/L.

#### Assessment of covariates

The possible covariates extracted include age (years), sex, race, education, marital status, family poverty-to-income ratio (PIR), smoking, drinking, physical activity, duration of arthritis (years), diabetes, hypertension, dyslipidemia, CVD, osteoporosis, fracture, chronic kidney disease (CKD), cancer, body mass index (BMI, kg/m^2^), obese, white blood cell count (WBC, 1,000 cells/μL), alanine aminotransferase (ALT, U/L), aspartate aminotransferase (AST, U/L), antirheumatics, non-steroidal anti-inflammatory agents, glucocorticoid, and immunosuppressive agents. The definition of CVD was based on self-reported questions about whether participants had ever been told they had angina, heart failure, heart attack, coronary heart disease, stroke, congestive heart failure, or if they were using medication for any of these conditions. Hypertension was defined as a self-reported physician diagnosis, a systolic blood pressure (SBP) of 130 mm Hg or higher, a diastolic blood pressure (DBP) of 80 mm Hg or higher, or the use of medication. Diabetes was defined as a self-reported physician diagnosis, HbA1c ≥ 6.5%, fasting glucose ≥126 mg/dL, or the use of glucose-lowering medications. Dyslipidemia was defined as total cholesterol ≥240 mg/dL, LDL ≥ 160 mg/dL, or the use of lipid-lowering medications. Osteoporosis was diagnosed based on a self-reported history or a femoral neck bone mineral density (BMD) T-score ≤ −2.5. CKD was defined as eGFR <60 mL/min/1.73 m^2^ calculated using the CKD-EPI equation.

### Statistical analysis

Considering the complex sampling design of NHANES, all analyses applied the appropriate weights for the NHANES samples. The number of cases and the composition ratio (*n* [%]) were used to describe the categorical data, and comparisons between two groups were made using the chi-square test. Continuous variables were represented as the weighted mean ± standard error (SE), and a *t*-test was used to compare the groups. Multiple imputation was performed for variables with missing values, and sensitivity analysis was performed on the data before and after interpolation (Supplementary Table S1).

The associations between EASIX and GGT with the risk of all-cause mortality were analyzed using weighted multivariate Cox regression models to estimate hazard ratios (HRs) and 95% confidence interval (CI). Model 1 adjusted for demographic factors, including age, race, education, marital status, and PIR. Model 2 further adjusted for complications, including diabetes, hypertension, dyslipidemia, CVD, osteoporosis, fracture, CKD, and cancer, in addition to the variables in Model 1. Model 3 adjusted for all covariates that were identified in the weighted univariate Cox analysis (*p* < 0.05, Supplementary Table S2), such as age, race, education, marital status, PIR, physical activity, duration of arthritis, diabetes, hypertension, dyslipidemia, CVD, osteoporosis, fracture, CKD, cancer, and glucocorticoid. Notably, a moderated analysis of GGT (moderator) on the connection between EASIX and all-cause mortality among patients with RA was conducted. Three models were developed by treating the GGT, EASIX, and GGT × EASIX as the independent variables and all-cause mortality as the dependent variable. Model 1: Adjusted for age, race, education, marital status, and PIR. Model 2: Adjusted for diabetes, hypertension, dyslipidemia, CVD, osteoporosis, fracture, CKD, and cancer, in addition to the variables in Model 1. Model 3: Adjusted for age, race, education, marital status, PIR, physical activity, duration of arthritis, diabetes, hypertension, dyslipidemia, CVD, osteoporosis, fracture, CKD, cancer, and glucocorticoid.

Additionally, subgroup analysis was performed based on age, duration of arthritis, diabetes, and hypertension. Multicollinearity among covariates was assessed using variance inflation factors (VIF), with all variables demonstrating VIF values of less than 5, indicating no significant collinearity. A *p*-value below 0.05 was considered statistically significant. All analyses were performed using SAS 9.4 software (SAS Institute, Inc., Cary, NC, United States).

## Results

### Population characteristics

A total of 2,543 RA individuals, with a mean age of 57.89 (0.38) years, were included in this study, comprising 1,047 (40.92%) males and 1,496 (59.08%) females. A total of 867 individuals developed all-cause mortality over a mean follow-up time of 122.86 ± 3.29 months. The characteristics of the research population according to survival status are shown in Table 1. Compared to survivors, participants in the all-cause mortality group were more likely to be older and non-Hispanic white, had lower education levels and PIR, a longer duration of arthritis, and higher prevalence of diabetes, hypertension, dyslipidemia, CVD, osteoporosis, fracture, CKD, and cancer (*p* < 0.05).

**Table 1:** Population characteristics.

Variables	Total (*n* = 2,543)	Alive (*n* = 1,676)	Dead (*n* = 867)	*p*
EASIX, mean (SE)	0.58 (0.01)	0.53 (0.01)	0.70 (0.03)	<0.001
EASIX, *n* (%)				<0.001
<0.476	1,210 (50.02)	865 (53.41)	345 (42.28)	
≥0.476	1,333 (49.98)	811 (46.59)	522 (57.72)	
GGT, U/L, mean (SE)	34.90 (1.21)	33.29 (1.49)	38.56 (2.07)	0.041
GGT, U/L, *n* (%)				0.485
<23	1,283 (50.59)	852 (51.16)	431 (49.28)	
≥23	1,260 (49.41)	824 (48.84)	436 (50.72)	
Age, years, mean (SE)	57.89 (0.38)	54.21 (0.39)	66.27 (0.63)	<0.001
Age, years, *n* (%)				<0.001
<60	1,011 (53.54)	843 (63.97)	168 (29.80)	
≥60	1,532 (46.46)	833 (36.03)	699 (70.20)	
Sex, *n* (%)				0.484
Male	1,047 (40.92)	653 (40.33)	394 (42.27)	
Female	1,496 (59.08)	1,023 (59.67)	473 (57.73)	
Race, *n* (%)				<0.001
Non-Hispanic White	1,066 (67.15)	605 (63.18)	461 (76.18)	
Non-Hispanic Black	720 (15.83)	500 (16.74)	220 (13.77)	
Others	757 (17.02)	571 (20.08)	186 (10.04)	
Education, *n* (%)				<0.001
High school and below	1,555 (54.41)	934 (49.64)	621 (65.27)	
University and above	988 (45.59)	742 (50.36)	246 (34.73)	
Marital status, *n* (%)				<0.001
Married	1,269 (55.43)	876 (58.82)	393 (47.70)	
Never married	185 (6.51)	140 (7.40)	45 (4.48)	
Others	1,089 (38.06)	660 (33.77)	429 (47.81)	
PIR, *n* (%)				<0.001
≤1.3	1,017 (30.98)	657 (29.12)	360 (35.23)	
1.3–3.5	962 (37.21)	608 (35.30)	354 (41.55)	
>3.5	564 (31.81)	411 (35.58)	153 (23.22)	
Smoking, *n* (%)				0.024
No	1,148 (41.84)	795 (43.71)	353 (37.57)	
Yes	1,395 (58.16)	881 (56.29)	514 (62.43)	
Drinking, times/week, *n* (%)				0.061
<5	2,387 (92.94)	1,593 (93.80)	794 (90.99)	
≥5	156 (7.06)	83 (6.20)	73 (9.01)	
Physical activity, MET*minutes/week, *n* (%)				<0.001
<450	290 (11.61)	194 (11.60)	96 (11.63)	
≥450	1,120 (47.85)	880 (55.86)	240 (29.62)	
Unknown	1,133 (40.54)	602 (32.53)	531 (58.75)	
Duration of arthritis, years, mean (SE)	14.00 (0.36)	12.89 (0.39)	16.53 (0.67)	<0.001
Diabetes, yes, *n* (%)	799 (23.99)	479 (20.66)	320 (31.58)	<0.001
Hypertension, yes, *n* (%)	1830 (65.71)	1,112 (59.68)	718 (79.41)	<0.001
Dyslipidemia, yes, *n* (%)	2068 (81.00)	1,337 (78.56)	731 (86.57)	<0.001
CVD, yes, *n* (%)	1,090 (38.68)	614 (32.78)	476 (52.10)	<0.001
Osteoporosis, *n* (%)				<0.001
No	1701 (65.16)	1,038 (60.83)	663 (75.02)	
Yes	296 (11.97)	156 (9.28)	140 (18.07)	
Unknown	546 (22.87)	482 (29.89)	64 (6.91)	
Fracture, yes, *n* (%)	317 (14.52)	172 (12.46)	145 (19.21)	0.003
CKD, yes, *n* (%)	644 (20.32)	305 (13.84)	339 (35.06)	<0.001
Cancer, yes, *n* (%)	373 (15.81)	212 (13.13)	161 (21.92)	<0.001
BMI, kg/m^2^, mean (SE)	30.28 (0.21)	30.74 (0.25)	29.24 (0.33)	<0.001
Obese, yes, *n* (%)	1,196 (46.01)	833 (48.14)	363 (41.17)	0.022
WBC, 1,000 cells/μL, mean (SE)	7.42 (0.07)	7.42 (0.09)	7.45 (0.11)	0.823
AST, U/L, mean (SE)	25.55 (0.32)	25.05 (0.36)	26.70 (0.83)	0.090
ALT, U/L, mean (SE)	25.01 (0.41)	25.18 (0.57)	24.62 (0.97)	0.659
Antirheumatics, yes, *n* (%)	215 (10.51)	151 (11.42)	64 (8.44)	0.050
Non-steroidal anti-inflammatory agents, yes, *n* (%)	356 (14.22)	248 (14.96)	108 (12.53)	0.207
Glucocorticoid, yes, *n* (%)	174 (7.06)	99 (6.44)	75 (8.47)	0.150
Immunosuppressive agents, yes, *n* (%)	156 (7.41)	114 (8.20)	42 (5.59)	0.075
Follow-up time, years, mean (SE)	122.86 (3.29)	129.35 (4.15)	108.08 (3.97)	<0.001

EASIX, endothelial activation and stress index; GGT, gamma-glutamyl transferase; PIR, poverty-to-income ratio; CVD, cardiovascular diseases; CKD, chronic kidney disease; BMI, body mass index; WBC, blood cell count; ALT, alanine aminotransferase; AST, aspartate aminotransferase; SE, standard error.

### Associations of EASIX and GGT with all-cause mortality

**Figure 1:**
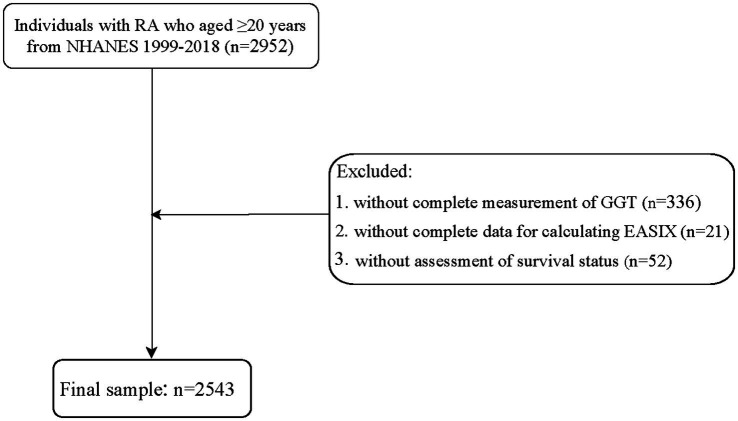
Flowchart of participant inclusion.

Table 2 presents the Cox regression models examining the associations between EASIX and GGT with all-cause mortality among patients with RA. In Model 1 adjusted for demographic factors, we found that higher EASIX (≥0.476) had an independent positive association with all-cause mortality risk (HR = 1.45, 95%; CI: 1.20–1.75; *p* < 0.001). After further adjusting the demographic factors and complications, the higher EASIX was associated with a 38% increased risk of all-cause mortality compared to those with lower EASIX (HR = 1.38; 95% CI: 1.14–1.68; *p* < 0.001). After fully adjusting for potential confounding factors, higher EASIX (≥0.476) remained significantly associated with all-cause mortality (HR = 1.42; 95% CI: 1.18–1.73; *p* < 0.001). However, three Cox regression models revealed that the associations between GGT and all-cause mortality were not significant (all *p* > 0.05). When analyzed as continuous variables, each 1-SD (or a 1 standard deviation increase) increase in EASIX was associated with an 18% higher risk of all-cause mortality (HR = 1.18; 95% CI: 1.06–1.31; *p* = 0.002), while GGT showed no significant association (HR = 1.04; 95% CI: 0.95–1.14; *p* = 0.387). Competing risks analysis accounting for cardiovascular mortality yielded consistent results (sHR = 1.39; 95% CI: 1.15–1.69), supporting the primary findings. The absolute 10-year mortality risk increased by 4.8% (12.3% vs. 17.1%) in high vs. low EASIX groups. EASIX-mortality associations were stronger in RA vs. non-RA participants (HR = 1.42 vs. 1.18; *p*_interaction_ = 0.032), underscoring RA-specific risks (see Figure 1).

**Table 2:** Associations of EASIX and GGT with all-cause mortality.

Variables	Model 1		Model 2		Model 3	
	HR (95% CI)	*P*	HR (95% CI)	*p*	HR (95% CI)	*p*
EASIX						
<0.476	Reference		Reference		Reference	
≥0.476	1.45 (1.20–1.75)	<0.001	1.38 (1.14–1.68)	<0.001	1.42 (1.18–1.73)	<0.001
GGT						
<23 U/L	Reference		Reference		Reference	
≥23 U/L	1.10 (0.93–1.29)	0.260	0.99 (0.85–1.17)	1.000	0.99 (0.85–1.17)	0.986

EASIX, endothelial activation and stress index; GGT, gamma-glutamyl transferase; HR, hazard ratio; CI, confidence interval; Model 1: Adjusted age, race, education, marital status, and poverty-to-income ratio (PIR); Model 2: Adjusted age, race, education, marital status, PIR, diabetes, hypertension, dyslipidemia, cardiovascular diseases (CVD), osteoporosis, fracture, chronic kidney disease (CKD), and cancer; Model 3: Adjusted age, race, education, marital status, PIR, physical activity, duration of arthritis, diabetes, hypertension, dyslipidemia, CVD, osteoporosis, fracture, CKD, cancer, and glucocorticoid.

### Moderated analysis of GGT

**Figure 2:**
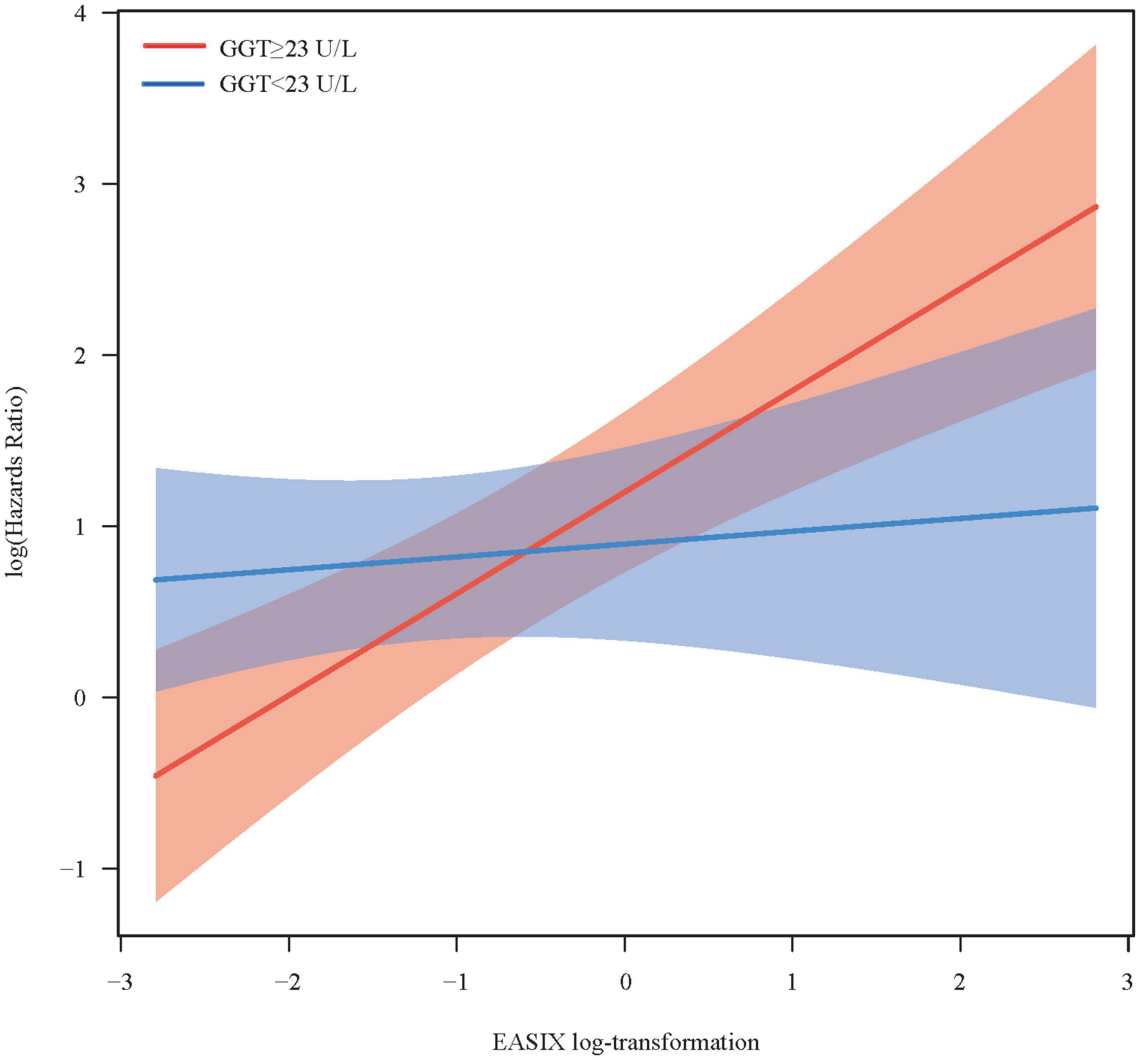
Conditional effect of endothelial activation and stress index on all-cause mortality across different gamma-glutamyl transferase concentrations.

The moderating role of GGT in the relationship between EASIX and all-cause mortality is shown in Table 3. After fully adjusting for potential confounding factors, the findings indicated that higher GGT levels resulted in an increased correlation between EASIX and all-cause mortality among patients with RA (*p* = 0.013). The association between EASIX and the risk of all-cause mortality exhibited varying patterns depending on GGT levels. More specifically, when GGT concentrations were below 23 U/L, the risk of all-cause mortality remained relatively stable as EASIX increased. However, when GGT concentrations were ≥23 U/L, a rapid increase in the risk of all-cause mortality was observed, accompanied by a rise in EASIX (Figure 2). In addition, as shown in Supplementary Table S3, no significant correlation was observed between higher EASIX and all-cause mortality in the fully adjusted model under low GGT concentrations. However, under high GGT concentrations, higher EASIX was associated with an increased risk of all-cause mortality, using the lower EASIX group as the reference (HR = 1.86; 95% CI: 1.38–2.50; *p* < 0.001). The adjusted effect size was also higher in the high GGT concentration group than in the low GGT concentrations group (HR = 1.86 vs. 1.13). These findings indicate that GGT plays a moderating role in the relationship between EASIX and all-cause mortality. In other words, high GGT levels may increase the risk of EASIX-related all-cause mortality of patients with RA.

**Table 3:** Moderated analysis of GGT.

Variables	Model 1		Model 2		Model 3	
	HR (95% CI)	*p*	HR (95% CI)	*p*	HR (95% CI)	*p*
EASIX	1.15 (0.88–1.51)	0.295	1.11 (0.87–1.42)	0.389	1.13 (0.89–1.45)	0.315
GGT	0.85 (0.65–1.10)	0.221	0.78 (0.61–0.99)	0.050	0.77 (0.60–0.99)	0.040
EASIX × GGT	1.56 (1.07–2.28)	0.021	1.53 (1.08–2.18)	0.017	1.57 (1.10–2.22)	0.013

EASIX, endothelial activation and stress index; GGT, gamma-glutamyl transferase; HR, hazard ratio; CI, confidence interval; Model 1: adjusted age, race, education, marital status, and poverty-to-income ratio (PIR); Model 2: adjusted age, race, education, marital status, PIR, diabetes, hypertension, dyslipidemia, cardiovascular diseases (CVD), osteoporosis, fracture, chronic kidney disease (CKD), and cancer; Model 3: adjusted age, race, education, marital status, PIR, physical activity, duration of arthritis, diabetes, hypertension, dyslipidemia, CVD, osteoporosis, fracture, CKD, cancer, and glucocorticoid.

### Subgroup analysis

We conducted a stratified analysis to assess the moderating role of GGT in the relationship between EASIX and all-cause mortality among patients with RA across different subgroups. As shown in Table 4, GGT may play a moderating role between EASIX and all-cause mortality among RA patients aged 60 years or older (*p* = 0.007), with a history of arthritis exceeding 5 years (*p* = 0.040), or diagnosed with diabetes (*p* = 0.009) or hypertension (*p* = 0.016). Additionally, high GGT levels may increase the risk of EASIX-related all-cause mortality in these specific populations (Supplementary Table S4).

**Table 4:** Subgroup analysis.

Subgroups	HR (95% CI)	*P*	HR (95% CI)	*P*
Subgroup I: age	<60 years (*n* = 1,011)		≥60 years (*n* = 1,532)	
EASIX	1.22 (0.62–2.41)	0.557	1.25 (0.95–1.65)	0.116
GGT	0.96 (0.62–1.48)	0.839	0.63 (0.45–0.89)	0.008
EASIX × GGT	1.37 (0.58–3.25)	0.468	1.74 (1.17–2.60)	0.007
Subgroup II: duration of arthritis	<5 years (*n* = 694)		≥5 years (*n* = 1,849)	
EASIX	1.42 (0.84–2.41)	0.190	1.08 (0.80–1.46)	0.603
GGT	0.63 (0.35–1.15)	0.133	0.83 (0.61–1.13)	0.244
EASIX × GGT	1.37 (0.63–2.98)	0.419	1.57 (1.02–2.42)	0.040
Subgroup III: Diabetes	No (*n* = 1,744)		Yes (*n* = 799)	
EASIX	1.20 (0.88–1.62)	0.244	1.01 (0.70–1.44)	0.985
GGT	0.85 (0.65–1.12)	0.246	0.61 (0.37–1.02)	0.058
EASIX × GGT	1.27 (0.83–1.95)	0.265	2.13 (1.21–3.75)	0.009
Subgroup IV: hypertension	No (*n* = 713)		Yes (*n* = 1,830)	
EASIX	1.34 (0.68–2.65)	0.396	1.07 (0.82–1.41)	0.609
GGT	0.80 (0.48–1.31)	0.368	0.75 (0.56–0.99)	0.051
EASIX × GGT	1.38 (0.55–3.52)	0.491	1.63 (1.10–2.42)	0.016

EASIX, endothelial activation and stress index; GGT, gamma-glutamyl transferase; HR, hazard ratio; CI, confidence interval; adjusted age (not adjusted in subgroup I), race, education, marital status, poverty-to-income ratio, physical activity, duration of arthritis (not adjusted in subgroup II), diabetes (not adjusted in subgroup III), hypertension (not adjusted in subgroup IV), dyslipidemia, cardiovascular diseases, osteoporosis, fracture, chronic kidney disease, cancer, and glucocorticoid.

## Discussion

This retrospective cohort study investigated not only the relationship between EASIX and the risk of all-cause mortality in patients with RA but also the moderating effect of GGT levels on all-cause mortality related to EASIX. The findings showed that higher EASIX was significantly associated with an increased risk of all-cause mortality among patients with RA. GGT moderated the relationship between EASIX and all-cause mortality.

Endothelial dysfunction plays a pivotal role in the pathogenesis of RA. It is widely acknowledged that inflammation, a characteristic feature of RA, triggers endothelial cell activation. The activated endothelial cells contribute to atherosclerosis by upregulating leukocyte adhesion molecules (7). Thus, certain biomarkers associated with endothelial dysfunction were deemed to possess prognostic significance for patients diagnosed with RA. Kurosaka and colleagues reported that vascular endothelial growth factor (VEGF) serves as a significant indicator of RA activity. At the same time, serum angiopoietin-1 (Ang-1) levels may provide valuable insights into sustained arthritis through the maintenance of newly formed vessels (20). In the study of López-Mejías et al., angiopoietin-2 (Angpt-2) levels were found to be associated with disease severity and CVD development in RA patients (21). Nonetheless, assessing endothelial dysfunction in clinical practice presents a considerable challenge, as there are currently no standard blood tests available for the direct evaluation of this condition. EASIX, initially described by Luft et al., is calculated using LDH, creatinine, and platelet levels and was developed to evaluate the prognostic significance in patients with acute graft-versus-host disease (GVHD) after allogeneic stem cell transplantation (1). Serum LDH, creatinine, and platelet levels are readily available in routine clinical practice and are also considered relevant parameters related to endothelial pathology (22). Currently, EASIX, as an endothelial dysfunction-related marker, has demonstrated clinical utility in evaluating the prognosis of various other diseases (22, 23). However, limited research has been conducted on the association between EASIX and prognosis in RA patients. Our study effectively fills this research gap by presenting new findings, indicating that higher EASIX may be a risk factor for all-cause mortality in patients with RA.

The GGT is an enzyme present in the kidneys, liver, pancreas, spleen, and vascular endothelium of the body, and it serves as a reliable biomarker for assessing hepatic function (24). Accumulating evidence suggests that GGT may be linked with the occurrence and progression of various diseases (24–26). Higher GGT levels may reflect inflammation-related oxidative stress (27) or can be attributed to the presence of inflammatory cytokines (28). In this study, no significant association was found between GGT and all-cause mortality in patients with RA. However, it is noteworthy that our study revealed a significant moderating effect of GGT on the association between EASIX and all-cause mortality. Specifically, elevated GGT levels may augment the risk of all-cause mortality related to EASIX in patients with RA. The presence of elevated GGT levels can lead to an imbalance between reactive oxygen species and antioxidant systems, resulting in oxidative stress. This oxidative stress is also a significant contributor to the development of endothelial dysfunction (11, 29). In addition, stratified analysis demonstrated the robustness of GGT as a moderating factor between EASIX and all-cause mortality in RA patients aged 60 years or older, those with a history of arthritis exceeding 5 years, or those diagnosed with diabetes or hypertension. The stronger moderating effect of GGT in older patients (≥60 years) may reflect age-related endothelial senescence and cumulative oxidative stress. Similarly, diabetes and hypertension likely amplify GGT’s role by exacerbating systemic inflammation and endothelial injury. The association in patients with longer RA duration aligns with the premise that chronic inflammation accelerates endothelial dysfunction, rendering EASIX more sensitive to oxidative stress markers, such as GGT. GGT catalyzes the breakdown of extracellular glutathione, releasing pro-oxidant cysteine–glycine dipeptides that generate reactive oxygen species (ROS) (30). In RA, systemic inflammation synergizes with GGT-mediated oxidative stress, accelerating endothelial apoptosis and thrombotic microangiopathy—key pathways linking EASIX to mortality (31). While GGT is a biomarker of oxidative stress rather than a direct therapeutic target, interventions that mitigate systemic oxidation (e.g., antioxidants and lifestyle modifications) may attenuate GGT-associated risks. More evidence is required to elucidate the underlying pathophysiological pathways of GGT in its moderating role.

Our study is the first to reveal a positive association between EASIX and the risk of all-cause mortality in patients with RA, while also clarifying the moderating effect of GGT levels on the association between all-cause mortality and EASIX. This finding not only expands the scope and significance of EASIX in clinical practice but also emphasizes the importance of controlling the GGT levels to reduce the risk of EASIX-related all-cause mortality in patients with RA. Nevertheless, our study also has certain limitations. First, this is a single-center study, which may introduce potential selection bias. Therefore, caution must be exercised when attempting to generalize these findings. Second, we only had single values of serum GGT levels and EASIX available, and the dynamic changes were not further assessed. Third, EASIX and GGT were measured only at baseline, and dynamic changes during follow-up (e.g., treatment effects or disease progression) were not captured. Future studies should incorporate longitudinal measurements to assess temporal relationships. Fourth, despite rigorous adjustment for covariates, residual confounding inherent in observational studies cannot be excluded. For example, unmeasured factors, such as genetic predisposition or environmental exposures, may influence both endothelial dysfunction and mortality.

Fifth, the generalizability to RA-specific cohorts requires validation, as NHANES lacks granular data on disease activity or treatment response. Sixth, the NHANES mortality data captures only all-cause mortality, without cause-specific classifications (e.g., cardiovascular, malignancy, or infection-related deaths). Future studies with cause-specific mortality data are needed to explore differential associations. Finally, despite our efforts to adjust for possible confounders, there still exist confounding variables, such as detailed medication treatment information, that could not be incorporated into the analysis due to limitations in the NHANES database.

## Conclusion

High EASIX may be positively associated with all-cause mortality in patients with RA, and this association was significantly enhanced by higher GGT levels.

## Data Availability

Publicly available datasets were analyzed in this study. This data can be found at: NHANES database, https://wwwn.cdc.gov/nchs/nhanes/.
