# Supplementary material for: Relationship between endothelial activation and stress index and all-cause mortality in rheumatoid arthritis patients: a moderating effect of gamma-glutamyl transferase
Source: Front Nutr. 2025 Apr 23;12:1554429. doi: 10.3389/fnut.2025.1554429 (PMC12055536; doi:10.3389/fnut.2025.1554429)
Supplement: Supplementary file 1 [file Table_1.docx]

**Supplemental Table 1** **Sensitivity analysis on the data before and** **after interpolation**

| **Variables** | **Before interpolation** | **After interpolation** | **Statistics** | ***P*** |
| --- | --- | --- | --- | --- |
| Education, n (%) |  |  | χ^2^=0.23 | 0.629 |
| High school and below | 1553 (54.42) | 1555 (54.41) |  |  |
| University and above | 986 (45.58) | 988 (45.59) |  |  |
| Marital status, n (%) |  |  | χ^2^=3.64 | 0.162 |
| Married | 1256 (55.44) | 1269 (55.43) |  |  |
| Never married | 184 (6.56) | 185 (6.51) |  |  |
| Others | 1074 (37.99) | 1089 (38.06) |  |  |
| PIR, n (%) |  |  | χ^2^=2.55 | 0.280 |
| ≤1.3 | 932 (31.28) | 1017 (30.98) |  |  |
| 1.3-3.5 | 878 (37.36) | 962 (37.21) |  |  |
| >3.5 | 516 (31.36) | 564 (31.81) |  |  |
| Smoking, n (%) |  |  | χ^2^=0.68 | 0.410 |
| No | 1146 (41.82) | 1148 (41.84) |  |  |
| Yes | 1394 (58.18) | 1395 (58.16) |  |  |
| Drinking, times /week, n (%) |  |  | χ^2^=1.36 | 0.244 |
| <5 | 2223 (92.82) | 2387 (92.94) |  |  |
| ≥5 | 147 (7.18) | 156 (7.06) |  |  |
| Duration of arthritis, years, Mean (S.E) | 13.98 (0.36) | 14.00 (0.36) | t=-0.89 | 0.372 |
| CKD n (%) |  |  | χ^2^=1.55 | 0.213 |
| No | 1870 (79.81) | 1899 (79.68) |  |  |
| Yes | 629 (20.19) | 644 (20.32) |  |  |
| Cancer, n (%) |  |  | χ^2^=2.08 | 0.150 |
| No | 2166 (84.17) | 2170 (84.19) |  |  |
| Yes | 373 (15.83) | 373 (15.81) |  |  |
| BMI, kg/m^2^, Mean (S.E) | 30.29 (0.21) | 30.28 (0.21) | t=0.07 | 0.947 |

PIR, poverty to income ratio; CKD, chronic kidney disease; BMI, body mass index.

**Supplemental Table 2 Identification of covariates by weighted univariate COX analysis**

| **Variables** | **HR (95%CI)** | ***P*** |
| --- | --- | --- |
| Age | 1.07 (1.06-1.08) | <0.001 |
| Gender |  |  |
| Male | Ref |  |
| Female | 0.92 (0.78-1.10) | 0.366 |
| Race |  |  |
| Non-Hispanic White | Ref |  |
| Non-Hispanic Black | 0.76 (0.61-0.94) | 0.013 |
| Others | 0.57 (0.44-0.74) | <0.001 |
| Education |  |  |
| High school and below | Ref |  |
| University and above | 0.68 (0.56-0.82) | <0.001 |
| Marital status |  |  |
| Married | Ref |  |
| Never married | 0.87 (0.56-1.36) | 0.546 |
| Others | 1.79 (1.52-2.11) | <0.001 |
| PIR |  |  |
| ≤1.3 | Ref |  |
| 1.3-3.5 | 0.97 (0.80-1.18) | 0.790 |
| >3.5 | 0.55 (0.42-0.70) | <0.001 |
| Smoking |  |  |
| No | Ref |  |
| Yes | 1.12 (0.93-1.33) | 0.225 |
| Drinking, times /week |  |  |
| <5 | Ref |  |
| ≥5 | 1.25 (0.92-1.69) | 0.146 |
| Physical activity, MET*minutes/week |  |  |
| <450 | Ref |  |
| ≥450 | 0.74 (0.55-1.01) | 0.055 |
| Unknown | 1.67 (1.22-2.30) | 0.002 |
| Duration of arthritis | 1.02 (1.01-1.03) | <0.001 |
| Diabetes |  |  |
| No | Ref |  |
| Yes | 1.85 (1.51-2.26) | <0.001 |
| Hypertension |  |  |
| No | Ref |  |
| Yes | 2.72 (2.16-3.44) | <0.001 |
| Dyslipidemia |  |  |
| No | Ref |  |
| Yes | 1.73 (1.36-2.21) | <0.001 |
| CVD |  |  |
| No | Ref |  |
| Yes | 2.17 (1.81-2.60) | <0.001 |
| Osteoporosis |  |  |
| No | Ref |  |
| Yes | 2.07 (1.58-2.72) | <0.001 |
| Unknown | 0.71 (0.48-1.05) | 0.087 |
| Fracture |  |  |
| No | Ref |  |
| Yes | 1.32 (1.01-1.73) | 0.046 |
| CKD |  |  |
| No | Ref |  |
| Yes | 3.07 (2.54-3.72) | <0.001 |
| Cancer |  |  |
| No | Ref |  |
| Yes | 1.85 (1.47-2.34) | <0.001 |
| Obese |  |  |
| No | Ref |  |
| Yes | 0.86 (0.70-1.04) | 0.117 |
| WBC | 1.01 (0.96-1.04) | 0.882 |
| AST | 1.01 (1.01-1.01) | 0.428 |
| ALT | 0.99 (0.98-0.99) | 0.224 |
| Antirheumatics |  |  |
| No | Ref |  |
| Yes | 0.87 (0.64-1.18) | 0.364 |
| Nonsteroidal anti-inflammatory agents |  |  |
| No | Ref |  |
| Yes | 0.76 (0.57-1.02) | 0.065 |
| Glucocorticoid |  |  |
| No | Ref |  |
| Yes | 1.43 (1.01-2.06) | 0.051 |
| Immunosuppressive agents |  |  |
| No | Ref |  |
| Yes | 0.87 (0.57-1.33) | 0.522 |

PIR, poverty to income ratio; CVD, cardiovascular diseases; CKD, chronic kidney disease; BMI, body mass index; WBC, blood cell count; ALT, alanine aminotransferase; AST, aspartate aminotransferase; HR, hazard ratio; CI, confidence interval.

**Supplemental Table 3 Association analysis between EASIX and all-cause mortality at different GGT levels**

| **Variables** | **Model 1******** | | **Model 2**** | | **Model 3**** | |
| --- | --- | --- | --- | --- | --- | --- |
|  | **HR (95% CI)** | ***P*** | **HR (95% CI)** | ***P*** | **HR (95% CI)** | ***P*** |
| GGT<23 U/L (n=1283) |  |  |  |  |  |  |
| EASIX<0.476 | Ref |  | Ref |  | Ref |  |
| EASIX≥0.476 | 1.14 (0.86-1.49) | 0.362 | 1.11 (0.84-1.47) | 0.456 | 1.13 (0.85-1.49) | 0.408 |
| GGT≥23 U/L (n=1260) |  |  |  |  |  |  |
| EASIX<0.476 | Ref |  | Ref |  | Ref |  |
| EASIX≥0.476 | 1.90 (1.44-2.51) | <0.001 | 1.81 (1.35-2.44) | <0.001 | 1.86 (1.38-2.50) | <0.001 |

EASIX, endothelial activation and stress index; GGT, gamma-glutamyltransferase; HR, hazard ratio; CI, confidence interval;

Model 1 ** adjusted age, race, education, marital status, and poverty to income ratio (PIR);

Model 2 ** adjusted age, race, education, marital status, PIR, diabetes, hypertension, dyslipidemia, cardiovascular diseases (CVD), osteoporosis, fracture, chronic kidney disease (CKD), and cancer;

Model 3 ** adjusted age, race, education, marital status, PIR, physical activity, duration of arthritis, diabetes, hypertension, dyslipidemia, CVD, osteoporosis, fracture, CKD, cancer, and glucocorticoid.

**Supplemental Table 4 Subgroup analysis on the associations between EASIX and all-cause mortality at different GGT levels**

| **Subgroups** | **GGT<23 U/L** | | **GGT≥23 U/L** | |
| --- | --- | --- | --- | --- |
|  | **HR (95% CI)** | ***P*** | **HR (95% CI)** | ***P*** |
| Subgroup I: Age≥60 years | 1.24 (0.93-1.67) | 0.142 | 2.31 (1.64-3.26) | <0.001 |
| Subgroup II: Duration of arthritis≥5 years | 1.04 (0.75-1.45) | 0.803 | 1.86 (1.34-2.57) | <0.001 |
| Subgroup III: Diabetes=Yes | 0.97 (0.67-1.40) | 0.859 | 2.32 (1.34-4.03) | 0.003 |
| Subgroup IV: Hypertension=Yes | 1.06 (0.79-1.43) | 0.700 | 1.89 (1.38-2.61) | <0.001 |

EASIX, endothelial activation and stress index; GGT, gamma-glutamyltransferase; HR, hazard ratio; CI, confidence interval;

Adjusted age (not adjusted in Subgroup I), race, education, marital status, poverty to income ratio, physical activity, duration of arthritis (not adjusted in Subgroup II), diabetes (not adjusted in Subgroup III), hypertension (not adjusted in Subgroup IV), dyslipidemia, cardiovascular diseases, osteoporosis, fracture, chronic kidney disease, cancer, and glucocorticoid.
